# Editorial: Role of carcinoembryonic antigen-related cell adhesion molecules in pathogen responses, tumorigenicity, and immune modulation

**DOI:** 10.3389/fimmu.2024.1388453

**Published:** 2024-03-05

**Authors:** Vishal Khairnar, Vu N. Ngo

**Affiliations:** ^1^ Cytovia Therapeutics, Natick, MA, United States; ^2^ Department of Systems Biology, Beckman Research Institute of City of Hope Comprehensive Cancer Center, Duarte, CA, United States

**Keywords:** CEA, CEACAMs, infectious diseases, cancer, glycoproteins

CEACAMs, short for carcinoembryonic antigen-related cell adhesion molecules, form a diverse family of twelve membrane glycoproteins. They are anchored to the apical surface of epithelial and endothelial cells, as well as on neutrophils, macrophages, and lymphocytes (1). Each CEACAM molecule possesses an extracellular immunoglobulin (Ig) variable (V)-like domain, enabling both homophilic interactions with self and heterophilic interactions with other CEACAM molecules (2). CEACAMs also serve as docking sites for bacterial and viral pathogens, thereby aiding infection processes (3–5). Extensive research spanning decades has uncovered a wide range of functions for the CEACAM family, encompassing fundamental cellular processes like migration, angiogenesis, proliferation, apoptosis, and immune cell regulation to pathological conditions such as infectious diseases and cancer (6–8). Advancements in research methodologies, including the development of specific monoclonal antibodies and animal models (9–13), have continued to improve our understanding of the role of CEACAM family in both health and diseases, as well as its clinical utility. Despite clear clinical implications, several key areas remain inadequately explored, including detailed signaling mechanisms associated with individual molecules and the functions of other less investigated CEACAMs (e.g., CEACAM4, CEACAM7, CEACAM8, CEACAM16, CEACAM18, CEACAM19, CEACAM20, and CEACAM21).

The Research Topic aims to maintain focus on the significance of this family of molecules. It includes six articles, comprising three Reviews and three Original Research articles. Recent data are used to review the notable interactions between CEACAMs and pathogens, as well as the pivotal roles of CEACAM1. In addition, the ongoing importance of CEACAM1 and CEACAM5 (CEA) as tumor biomarkers in prostate and colorectal cancers is highlighted in the Original Research articles.

The CEACAM family, serving as a binding receptor for various microbial pathogens, is not only utilized by these microorganisms as a docking site for infection but also as a means to evade the host cell response. In their review, Nguyen et al. delve into the effects of *Helicobacter pylori* adhesin HopQ binding to CEACAM receptors in human gastric cells. The review particularly emphasizes recent structural studies elucidating the interaction between the *Helicobacter* virulence factor HopQ and CEACAMs, along with the resulting impact on the inflammatory response in the gastric epithelia and other immune cells. Structural insights into the HopQ-CEACAM complex highlight *H. pylori*’s ability to shift the equilibrium between dimeric and monomeric CEACAM1, favoring monomeric CEACAM1 interaction with HopQ. Beyond regulating epithelial cell responses, the HopQ-CEACAM interaction also influences the response and activity of diverse immune cell populations. This includes modulation of chemokine secretion in neutrophils and macrophages, hindrance of IFNγ secretion by CD4^+^ T cells, and suppression of cytotoxic activity of NK cells and CD8^+^ T cells. Thus, through the HopQ-CEACAM interaction on immune cells, *H. pylori* can extend infection in gastric cells by limiting the host inflammatory response.

Continuing the exploration of pathogen-CEACAM interactions, Sheikh and Fleckenstein conduct a review on the interactions of pathogenic *Escherichia coli* with CEACAMs. Several studies have revealed diverse engagement strategies with CEACAMs employed by various pathogen variants, also known as pathovars. In the case of the adherent-invasive *E. coli* (AIEC) pathovar, interaction with CEACAM6 occurs through the bacterial adhesin FimH. This interaction may induce CEACAM6 expression via inflammatory mediators, amplifying AIEC binding. For the diffusely adhering *E. coli* (DAEC) pathovar, a subset of the bacterial virulence factors, the Afa/Dr adhesins, bind to CEACAM1, CEACAM5, and CEACAM6 along gastrointestinal and urinary epithelia. These engagements with CEACAMs can enhance pathogen colonization by suppressing bacterial elimination through the host defense. In the context of the enterotoxigenic *E. coli* (ETEC) pathovar, primarily responsible for acute diarrheal illness, the production of heat-labile toxin leads to significantly increased expression of CEACAM5, CEACAM6, and CEACAM7. These, in turn, serve as crucial receptors for ETEC to establish their niche in the small intestine. Interestingly, the interaction with CEACAMs is not always advantageous to the pathogens, as CEACAMs can act as molecular decoys shedding from the gastrointestinal tract. While the innate defense by CEACAMs warrants further investigation, ongoing studies of pathogen-CEACAM interactions hold promises in enhancing our understanding and influencing the outcomes of these infections.

Beyond the intricate dynamics of their interactions with pathogens, the intriguing involvement of CEACAMs in cancer development remains insufficiently explored. Recent findings in this topic highlight the substantial impact of CEACAMs, particularly in contributing to cancer development and serving as a potential tumor biomarker in clinical settings. In a study by Kube-Golovin et al., the CEACAM1-L isoform and other markers of malignant transformation were found to be synergistically upregulated by the pro-inflammatory cytokines TNF-α and IFN-γ in an *in vitro* prostatitis model using normal prostate RWPE-1 cells. While CEACAM1 was implicated in this process, further investigation employing CEACAM1-deficient cells in such a system would strengthen our understanding of CEACAM1’s intriguing role in inflammation-induced prostate cancer. In other Original Research articles in this topic, Lozada-Martinez et al. identified a significant association between differences in CEA levels before and after chemotherapeutic treatments (CEA-delta) and other cancer stem cell markers in rectal cancer (RC). This suggests the potential use of CEA-delta as a clinical biomarker for tumor classification, staging, and prognosis of cancer stem cell-positive RC. Additionally, Xie et al. confirm CEA as an independent prognostic factor in colorectal cancer (CRC), highlighting its enhanced prognostic significance when used in conjunction with the TNM staging system. These findings underscore the multifaceted role of CEACAMs in cancer biology and their clinical implications.

Within the CEACAM family, CEACAM1 stands out as the sole member harboring two immunoreceptor tyrosine-based motifs capable of interacting with SRC homology 2 (SH2) domain-containing kinases or phosphatases, leading to diverse signaling outcomes. In their review, Götz et al. provide a comprehensive summary of clinical data concerning the involvement of CEACAM1 in cancer initiation and progression, alongside an overview of potential mechanisms and clinical applications. The review examines contrasting evidence regarding whether CEACAM1 acts as a tumor suppressor or a cancer driver, while also shedding light on the various mechanisms underlying CEACAM1 functions to reconcile conflicting findings in the literature. Moreover, the authors thoroughly examine the advantages and disadvantages of employing anti-CEACAM1 strategies for cancer therapy.

## Author contributions

VN: Writing – original draft. VK: Writing – review & editing.

## In memoriam

This Research Topic is dedicated to the memory of our late Guest Editor, Professor Bernhard B. Singer, University Hospital, Essen, Germany, who served as a source of inspiration and played a pivotal role during its development. His untimely passing in early January 2023 has been a profound loss to both the scientific community and the CEACAM field. As an exceptionally inspiring leader, Professor Bernhard B. Singer was devoted to advancing the field not just through his remarkable scientific contributions but also through his unwavering advocacy for science communication and his steadfast commitment to fostering young scientists. Undoubtedly, Professor Bernhard B. Singer’s legacy, along with his unfinished work and vision, will continue to inspire and drive us forward in our collective pursuit of knowledge through scientific inquiry.
